# Antimicrobial activity of *Terminalia arjuna* Wight & Arn.: An ethnomedicinal plant against pathogens causing ear infection

**DOI:** 10.1590/S1808-86942012000100011

**Published:** 2015-10-20

**Authors:** Kamal Rai Aneja, Chetan Sharma, Radhika Joshi

**Affiliations:** a Dr (Professor); b Research scholar (Kurukshetra); c Research scholar

**Keywords:** antimicrobial activity, ear pathogen, plant extract

## Abstract

Ear infection is one of the common diseases occurring throughout the world. Different etiological agents are responsible for ear infections.

**Aim:**

To assess the antimicrobial potential of *Terminalia arjuna* leaves and bark extracts against *Staphylococcus aureus*, *Acinetobacter sp.*, *Proteus mirabilis*, *Escherchia coli*, *Pseudomonas aeruginosa* and *Candida albicans*, pathogens causing ear infections and their comparison with locally available ear drops.

**Materials and Methods:**

Methanol, ethanol, acetone, aqueous (hot and cold) extracts from the leaves and bark of *T. arjuna* were tested for their antimicrobial activity.

**Results:**

Of the three organic solvents evaluated, acetonic leaf extract was found to be best against *S. aureus*. Organic bark extract showed almost equal inhibition of all tested Gram negative bacteria except *P. aeruginosa*. However, aqueous extract of *T. arjuna* bark exhibited good activity against *S. aureus*. All the extracts were unable to exhibit any antifungal activity.

**Conclusion:**

Organic extract obtained from the *T. arjuna* bark and leaves may be used to treat the bacterial ear pathogens especially *S. aureus*, which has shown greater inhibition zones than the herbal drops, however, we still need more detailed studies as *in vivo* testing and pharmacokinetics properties for their therapeutic utility in treating ear infections.

## INTRODUCTION

Ear infection is more common in children than adults, approximately 75% of children experience at least three or more ear infections during the first three years of life^1^. Ear infection is mainly caused by bacterial (*Pseudomonas aeruginosa, Staphylococcus aureus, S. epidermidis, Streptococcus pneumoniae, Escherichia coli, Proteus mirabilis)* and fungal pathogens (*Aspergillus niger, A. fumigatus, A. flavus, Candida albicans*)^2^, ^3^, ^4^, ^5^.

The clinical efficacy of many existing chemotherapeutic agents is being threatened by the emergence of multidrug-resistant pathogens. Bacteria naturally develop resistance to antimicrobial drugs. In recent years, however, the overuse and misuse of antibiotics has caused a growing number of staphylococcus bacteria to evolve into disease causing “superbugs” resistant to drugs like methicillin, vancomycin^6^, ^7^, ^8^. The increasing failure of chemotherapeutics and antibiotic resistance exhibited by pathogenic microbial infectious agents has led to the screening of several plants for their potential antimicrobial activity and development of new antimicrobials by drug companies^7^, ^9^. Herbs are staging a comeback and herbal 'renaissance' is happening all over the globe and according to WHO, 80% of the world's population relies on plant-based traditional medicines for their primary healthcare needs^10^, ^11^, ^12^.

Nature has bestowed India with rich flora that is widely distributed. Herbal medicines have been used for the treatment and cure of various diseases in the Indian traditional practiced methods such as Ayurveda, Unani and Siddha^13^, ^14^. *Terminalia arjuna* Wight & Arn., commonly called arjuna (family *Combretaceae*), is a deciduous and evergreen tree distributed throughout India and also found in Burma, Srilanka and Mauritius, growing up to a height of 60 to 90 feet. Leaves of arjuna are simple, oblong or elliptic with pale and dark green upper surface and pale brown lower surface. Flowers are bisexual, sessile and white arranged in short axillary spikes or in terminal pannicule. The bark is smooth, pinkish-grey from outside and flakes off in large, curved and rather flat pieces^15^, ^16^. The bark and leaves of this plant have been used in indigenous system of medicine for curing different diseases, the bark in the treatment for angina (hritshool),expectorant, antidysentric, purgative, laxative, leucoderma, anaemia, hyperhidrosis, asthama, tumors and other cardiovascular disorders^16^, ^17^ and leaves as a remedy for the treatment of ear ache^18^. The bark also possesses good anticancer, antiviral and antimicrobial activities^19^, ^20^.

Plants have limitless ability to synthesize secondary metabolites such as tannins, terpenoids, alkaloids, flavonoids, glycosides and phenols which have been found to have antimicrobial properties^11^, ^21^, ^22^. The leaves and bark of *T. arjuna* contain glycosides having cardio protective effect, flavanoids having antioxidant, anti-inflamatory, antimicrobial (luteolin), anti cancerous and lipid lowering effects, tannins responsible for astringent, wound healing, antioxidant, anti cancer, anti viral and antimicrobial activity. In addition to these phytocompounds bark also contains triterpenoids responsible mainly for cardio protective and antibacterial (arjunic acid, arjungenin and, arjunetin) effect^15^, ^16^, ^23^.

A wide variety of medicinal plants used traditionally have not yet been systematically investigated against various microbial pathogens^24^. Literature search reveals the conducting of a few studies on antibacterial and antifungal activity of various parts of this plant, such as bark^23^, ^25^, ^26^, leaves, root and fruit^15^, ^27^, ^28^. However, antimicrobial studies against pathogens causing ear infection are lacking. The present study was carried out to validate the antimicrobial potential of *T. arjuna* leaves and bark against the bacterial and fungal ear pathogens isolated from the local patients of Kurukshetra and their comparison with locally available ear drops with a view of searching a novel extract as a remedy for treating ear infections.

## MATERIAL AND METHODS

### Plant collection

The bark and leaves of *Terminalia arjuna* were collected from the trees alongside the roads of University of Kurukshetra, Haryana. The taxonomic identity of this plant was confirmed by Dr. B.D. Vashishta of Botany Department, Kurukshetra University, Kurukshetra.

### Extraction of plant material

The samples were carefully washed under running tap water followed by sterile distilled water and air dried at room temperature (40°C) for 4-5 days and then homogenized to a fine powder using a sterilized mixer grinder and stored in air tight bottles. Four different solvents namely ethanol, methanol, acetone and aqueous (hot and cold) were used for extraction. A 10 g amount of homogenized bark and leaves were separately soaked in conical flasks each containing100 ml of acetone, ethanol, methanol (95%) and sterile distilled water. Also the same amount (i.e. 10 g) of homogenized bark and leaves were immersed separately in 100 ml of hot sterile distilled water in conical flasks and allowed to stand for 30 min on a waterbath with occasional shaking followed by keeping all the flasks on rotary shaker at 200 rpm for 24 h^29^, ^30^, ^31^. Each preparation was filtered through a sterilized Whatman No. 1 filter paper and finally concentrated to dryness under vacuum at 40°C using rotaevaporator. The dried extract thus obtained was sterilized by overnight UV-irradiation and checked for sterility on nutrient agar plates and stored at 4°C in labeled sterile bottles until further use^14^, ^32^.

### Test microorganisms

Five bacteria namely *Staphylococcus aureus* (HM626197)* (Gram-positive)*, Acinetobacter* sp. (HM626198), *Proteus mirabilis* (HM626199)*, Escherchia coli* (HM626200)*, Pseudomonas aeruginosa* (HM626201) (Gram-negative) and one yeast, *Candida albicans* used in this study, were isolated from the patients having ear infection from the local ENT clinics of Kurukshetra. Bacterial strains were identified on the basis of staining, biochemical and molecular characteristics (16S rRNA sequencing)^33^ and yeast on the basis of staining, morphological and cultural characteristics^5^, ^34^, ^35^. The bacterial isolates were subcultured on Nutrient agar and yeast on Malt yeast agar and incubated aerobically at 37^o^C. The media were procured from Hi Media Laboratory Pvt. Ltd., Bombay, India.

### Ear drops

Three commonly prescribed ear drops by otolaryngologist, two allopathic Ciplox (antibacterial), Candid (antifungal), and a herbal ear drop Bilwa Tel (antimicrobial), used in this study, were procured from the local market of Kurukshetra.

### Screening for antimicrobial activity

The acetone, methanol, ethanol, hot and cold aqueous *T. arjuna* leaves and bark extracts were used for evaluation of the antimicrobial activity by the agar well diffusion method^36^, ^37^. In this method, pure isolate of each microbe was subcultured on the agar media plates at 37^0^C for 24 h. One plate of each microorganism was taken and a minimum of four colonies were touched with a sterile loop and transferred into normal saline (0.85%) under aseptic conditions. Density of each microbial suspension was adjusted equal to that of 10^6^ cfu/ml (standardized by 0.5McFarland standard) and used as the inoculum for performing agar well diffusion assay. One hundred microlitre (100μl) of inoculum of each test organism was spread onto the agar plates so as to achieve a confluent growth. The agar plates were allowed to dry and wells of 8mm were made with a sterile borer in the inoculated agar plates and the lower portion of each well was sealed with a little specific molten agar medium. The dried extracts were reconstituted in 20% dimethylsulphoxide (DMSO) for the bioassay analysis^38^. A 100μl volume of each extract was propelled directly into the wells (in triplicates) of the inoculated agar plates for each test organism. The plates were allowed to stand for 1hr for diffusion of the extract into the agar and incubated at 37^O^C for 24h.^32^, ^39^. Sterile DMSO (20%) served as the negative control and ciplox (for bacteria), candid (for fungi) and Bilwa tel (antimicrobial) ear drop served as the positive control. The antimicrobial activity, indicated by an inhibition zone surrounding the well containing the extract, was recorded if the zone of inhibition was greater than 8mm^40^. The experiments were performed in triplicates and the mean values of the diameter of inhibition zones with ± standard deviation were calculated.

### Determination of minimum inhibitory concentration (MIC)

MIC for each test organism was determined by following the modified agar well diffusion method. A twofold serial dilution of each extract was prepared by first reconstituting the dried extract (100 mg/ml) in 20% DMSO followed by dilution in sterile distilled water (1:1) to achieve a decreasing concentration range of 50mg/ml to 0.39mg/ml. A 100 μl volume of each dilution was introduced into wells (in triplicate) in the agar plates already seeded with 100μl of standardized inoculum (10^6^ cfu/ml) of the test microbial strain. All test plates were incubated aerobically at 37^o^C for 24 hrs and observed for the inhibition zones. MIC, taken as the lowest concentration of the test extract that completely inhibited the growth of the microbe, showed by a clear zone of inhibition (>8mm), was recorded for each test organism^32^, ^37^, ^41^, ^42^.

### Statistical analysis

All data were presented as mean ± SD (Standard deviation). Results were statistically evaluated using Dennett's T-test. P value less than 0.01 were considered significant.

## RESULTS

The antibacterial activity of *T. arjuna* bark and leaves extracts on the agar plates varied in different solvents. Of the ten extracts screened for antifungal activity, none of the extract showed the production of inhibition zone against *Candida albicans* thus lacking antifungal activity. Positive controls produced significantly sized inhibition zones against the tested bacteria and yeast, however, negative control produced no observable inhibitory effect against any of the test organism (Table 1).

**Table 1:** Antimicrobial activity of Terminalia arjuna leaves and bark extract on ear pathogens determined by agar well diffusion method.

Arjuna plant	Diameter of growth of inhibition zones (mm)					
	*Staphylococcus aureus*	*Pseudomonas aeruginosa*	*Proteus mirabilis*	*Escherchia coli*	*Acitenobacter Sp.*	*Candida albicans*
Leaves extracts (mg/ml)						
Methanol	25.6*^a^ ±0.57^b^	16.3±0.57	24.6±0.57	-	15±0	-
Ethanol	26.3±0.57	15.6±0.57	26±0	-	15.3±0.57	-
Acetone	28±0	16±0	27.6±0.57	-	16.6±0.57	-
Hot aqueous	-	-	-	-	-	-
Cold aqueous	-	-	-	-	-	-
Bark extracts (mg/ml)						
Methanol	23.6±0.57	-	15±0	15.6±0.57	17±0	-
Ethanol	26±0	-	14.6±0.57	15±0	16.3±0.57	-
Acetone	23.3±0.57	-	16.3±0.57	15.6±0.57	17.6±0.57	-
Hot aqueous	27.6±0 .57	-	-	-	-	-
Cold aqueous	26.3±0.57	-	-	-	-	-
DMSO	0	0	0	0	0	0
Ciplox ear drop	56.3±0.57	34±0	46.3±0.57	36±0	32.6±0.57	nt
Bilva tel ear drop	13.6±0.57	-	11.6±0.57	-	-	-
Candid ear drop	nt	nt	nt	nt	nt	21.3±0.57

- No activity, nt = not tested.

a Values, including diameter of the well (8mm), are means of three replicates

b ± Standard deviation. The data were analyzed by one way ANOVA followed by Dunnett's *t* test.

* *p*<0.01 compared to positive control.

**Figure 1:**
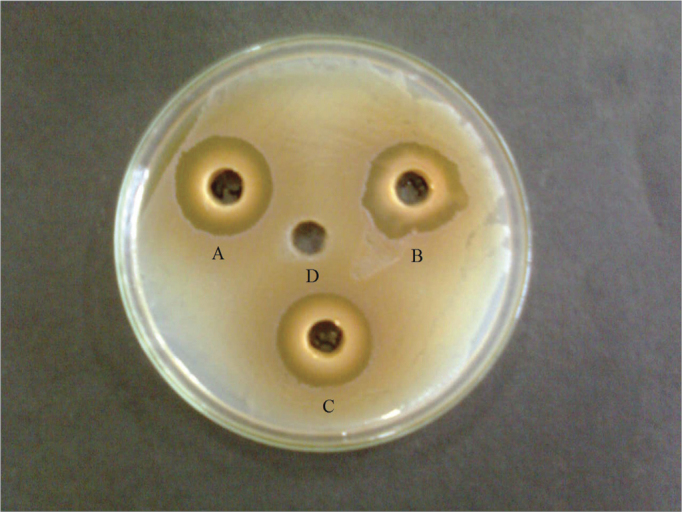
Zone of antibacterial inhibition of T. arjuna leaves organic extracts against S. aureus by acetonic (A), methanolic (B), ethanolic extract (C), and negative control DMSO (D).

A perusal of the data in Table 1 reveals that all the six organic solvent extracts of both leaves and bark possessed antibacterial activity against the four bacterial pathogens, of the five tested pathogens. The acetonic leaf extract was found to be most effective against *Staphylococcus aureus* (28mm) (Figure 1) followed by *Proteus mirabilis* (27.6mm), *Acinetobacter* sp (16.6mm) and *Pseudomonas aeruginosa* (16mm). The inhibiton zones produced by the methanolic and ethanolic extracts against *S. aureus* and *P. mirabilis* ranged between 24.6mm and 26.3 mm. *S. aureus* and *P. mirabilis* were found to be most sensitive pathogens which survived upto 1.56 mg/ml and 3.12 mg/ ml (Table 2), thus having an MIC of 3.12 mg/ml and 6.25 mg/ml, respectively. The inhibition zone produced by the organic leaf extracts against *P. aeruginosa* and *Acitenobacter* sp. ranged between 15mm and 16.6mm. *P. aeruginosa* and *Acitenobacter* sp. were found to be comparatively less sensitive as they survived up to 25mg/ml (methanolic and ethanolic extracts) and 12.5mg/ml (acetonic extract) thus having an MIC of 50 mg/ml and 25 mg/ml, respectively. No antibacterial activity was observed in aqueous and organic leaf extracts against *E. coli*.

**Table 2:** MIC of Terminalia arjuna leaves and bark extract on ear pathogens determined by modified agar well diffusion method.

Arjuna plant	MIC (mg/ml)				
	*Staphylococcus aureus*	*Pseudomonas aeruginosa*	*Proteus mirabilis*	*Escherchia coli*	*Acitenobacter* Sp.
Leaves extracts					
Methanol	6.25	50	6.25	nt	50
Ethanol	6.25	50	6.25	nt	50
Acetone	3.12	25	3.12	nt	25
Bark extracts					
Methanol	3.12	nt	50	25	25
Ethanol	1.56	nt	50	50	25
Acetone	3.12	nt	25	50	25
Hot aqueous	1.56	nt	nt	nt	nt
Cold aqueous	1.56	nt	nt	nt	nt

nt = not tested

**Figure 2:**
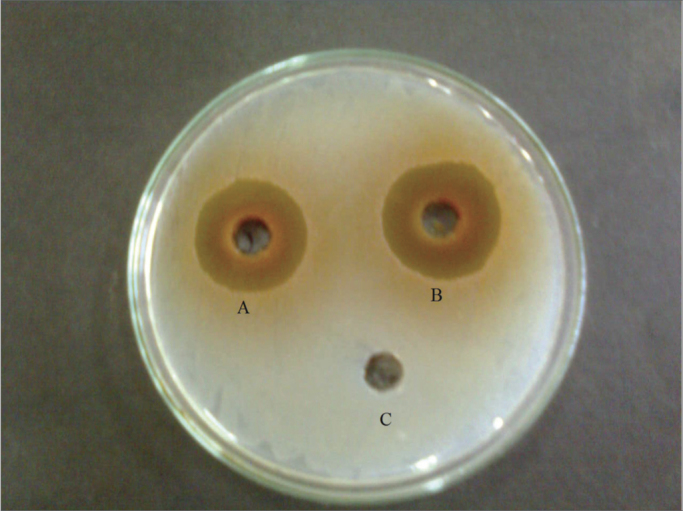
Zone of antibacterial inhibition of T. arjuna bark aqueous extracts against S. aureus by hot aqueous (A), cold aqueous extract (B), and negative control DMSO (C).

Among the tested bark extracts, all the five solvent extracts (organic and aqueous) showed antibacterial activity against *S. aureus* with highest zone of inhibition in hot aqueous extract (27.6mm) followed by cold aqueous extract (26.3mm) (Figure 2), ethanol extract (26mm), methanol and acetone extracts (23mm). *S. aureus* was again found to be the most sensitive pathogen which survived upto lowest concentration i.e.0.78 mg/ml in hot water, cold water and ethanol extracts (Table 2), thus having an MIC of 1.56 mg/ml followed by the methanolic and acetonic extracts 3mg/ml. The inhibition zones produced by the three organic solvents against *Acitenobacter* sp. ranged between 16.3mm and 17mm. *Acitenobacter* sp. was comparatively less sensitive as it survived upto 12.5 mg/ml, thus having an MIC of 25mg/ml. The zone of inhibition produced by the organic solvent bark extracts against *P. mirabilis* and *Escherchia coli* was almost equal and ranged between 14.6mm and 16.3mm. *P. mirabilis* survived up to 12.5mg/ml (acetone) and 25mg/ml (methanol and ethanol), thus having an MIC of 25mg/ml and 50 mg/ml respectively while *E. coli* survived upto 25mg/ml (ethanol and acetone) and 12.5mg/ml (methanol) thus having an MIC of 50 mg/ml and 25 mg/ml, respectively. Bark extracts, both organic and aqueous, however, lacked antibacterial activity against *P. aeruginosa*. All the obtained results are statistically significant as they showed (p < 0.001) as compared with control.

## DISCUSSION

The organic extracts of *T. arjuna* leaves possessed good activity against the four bacterial ear pathogens namely *Staphylococcus aureus, Pseudomonas aeruginosa, Proteus mirabilis*, *Acitenobacter sp.* and did not exhibit any activity against *E. coli.* Interestingly, the bark extract also showed good bioactivity against the four bacterial ear pathogens, however, *P. aeruginosa* was not found sensitive to the bark extracts. The antifungal activity against *C. albicans* was totally absent in the both organic and aqueous *T. arjuna* leaf and bark extracts. In earlier study, the root extracts of this plant had been shown to possess activity against *Aspergillus niger* and *Candida albican*^27^.

The aqueous leaves and bark extracts of *T. arjuna* lacked antifungal activity against *C. albicans* and antibacterial activity against the tested bacteria except a Gram positive bacterium, *S. aureus* that was found sensitive both to the hot and cold aqueous bark extracts. The limited spectrum of antimicrobial activity in the aqueous extracts may be due to three reasons: firstly the polarity of antibacterial compounds make them more readily extracted by organic solvents as compared to aqueous extract; secondly active compound may be present in insufficient amount in the crude extract to show activity with the dose level employed; and thirdly if the active principle is present in high quantities, there could be other constituents present in the extract exerting antagonistic effects of the bioactive compounds^43^.

Of the three organic extracts of leaves screened, the acetonic extract has been found to have a better antibacterial activity than the corresponding ethanolic and methanolic extracts (Table 1) thus substituting the findings of earlier workers^11^, ^37^, ^44^, ^45^ who rated acetone as the best solvent. The antibacterial activity exhibited by *T. arjuna* leaves and bark extracts against the bacterial ear pathogens might be due to the presence of secondary metabolites such as arjunic acid, arjungenin, arjunetin and luteolin which have been reported to be antimicrobial in nature^15^, ^16^, ^23^.

This study reveals that both the leaves and bark organic extracts of *T. arjuna* have broad spectrum antibacterial activity against the ear pathogens as visualized by the formation of inhibition zones of both Gram positive and Gram negative bacteria. Interestingly, all the organic extracts of this plant showed much more potent activity against the tested ear bacteria than that of standard herbal ear drop (Bilwa tel) thus having a great potential to be developed as a herbal ear drop to control the bacterial ear infections.

## CONCLUSIONS

Our results allow us to conclude that, all the tested *T. arjuna* extracts (in any of the tested preparation-acetone, ethanol or methanol) have shown good activity against both the Gram positive and Gram negative ear pathogens, the inhibition being higher in Gram positive bacteria than the Gram negative bacteria. This probably explains the use of this plant by the indigenous people against a number of infections since generations. As of now, little work has been done on the antimicrobial activity and plausible medicinal applications of the phytochemical compounds and hence extensive investigations are needed such as *in vivo* studies on this plant necessary to determine toxicity of the active constituents, their side effects, pharmacokinetics properties to exploit the bioactive principles, for therapeutic utility in treating the ear infections. The antibacterial activities can be enhanced if the active components are purified and adequate dosage determined for proper administration. At last, the need of the hour is the development of an effective phytocompound into an exploitable herbal product, which is devoid of side effects and drug resistance problem.

## Footnotes

* Nucleotide sequence of all the five bacteria have been submitted to GenBank database, which have provided GenBank accession number, HM626197-HM626201.
